# CuONPs/MWCNTs/carbon paste modified electrode for determination of tramadol: theoretical and experimental investigation

**DOI:** 10.1038/s41598-023-34569-y

**Published:** 2023-05-17

**Authors:** Razieh Razavi, Mahnaz Amiri, Kouros Divsalar, Alireza Foroumadi

**Affiliations:** 1 Department of Chemistry, Faculty of Science, University of Jiroft, Jiroft, Iran; 2 Neuroscience Research Center, Institute of Neuropharmacology, Kerman University of Medical Science, Kerman, Iran; 3 Department of Medicinal Chemistry, Faculty of Pharmacy, Drug Design & Development Research Center, The Institute of Pharmaceutical Sciences (TIPS), Tehran University of Medical Sciences, Tehran, Iran

**Keywords:** Analytical chemistry, Materials chemistry, Quantum chemistry, Nanoparticle synthesis

## Abstract

A practical technique was applied to fabricate CuO nanostructures for use as the electrocatalyst. The green synthesis of cupric oxide nanoparticles (CuO NPs) via co-precipitation is described in this paper using an aqueous extract of Origanum majorana as both reductant and stabilizer, accompanied by characterization via XRD, SEM, and FTIR. The XRD pattern revealed no impurities, whereas SEM revealed low agglomerated spherical particles. CuO nanoparticles and multi wall carbon nanotubes (MWCNTs) have been used to create a modified carbon paste electrode. Voltammetric methods were used to analyze Tramadol using CuONPs/MWCNT as a working electrode. The produced nanocomposite showed high selectivity for Tramadol analysis with peak potentials of ~ 230 mV and ~ 700 mV and Excellent linear calibration curves for Tramadol ranging from 0.08 to 500.0 µM with a correlation coefficient of 0.9997 and detection limits of 0.025. Also, the CuO NPs/MWCNT/CPE sensor shows an an appreciable sensitivity of 0.0773 μA/μM to tramadol. For the first time the B3LYP/LanL2DZ, quantum method was used to compute DFT to determine nanocomposites' connected energy and bandgap energy. Eventually, CuO NPs/CNT was shown to be effective in detecting Tramadol in actual samples, with a recovery rate ranging from 96 to 104.3%.

## Introduction

Tramadol is a synthetic opioid analgesic that works primarily on the central nervous system. It works through two fundamental mechanisms: agonistic binding to -opioid receptors and blocking norepinephrine and serotonin reuptake. Tramadol's pharmacokinetics, effectiveness, and safety qualities have made it a success in patients with moderate to severe chronic pain who take it three to four times a day. When compared to the usual form of Tramadol, extended-release Tramadol, a newly created modified-release tablet, would be favorable for the day-long duration and minor drug plasma variance^1,2^.

Tramadol is a substance that acts as a -agonist. [2-(dimethyl aminomethyl)-1(3-methoxyphenyl) cyclohexanol] is the chemical name. It is used to treat most forms of neuralgia, including trigeminal neuralgia, as well as moderate to severe pain. Several analytical techniques for determining Tramadol and other combination drugs have been published in the literature, including the spectrophotometric method^3,4^ and spectrophotometric and spectrofluorimetric approaches^5–7^.

Nanotechnology is now regarded as a cutting-edge subject of research that involves the creation of nanoparticles of various sizes, shapes, and chemical structures with a wide range of possible uses^8^. For the synthesis and design of nanoparticles, many procedures have been reported, including microwave irradiation^9^, photoreduction^10^, thermal breakdown^11^ and mechanical grinding^12^ but these procedures are mainly costly, energy-consuming, or hazardous to humans and the environment. Environmentally friendly methods should be implemented as a result. Green synthesis refers to the development of chemical and physical techniques that are environmentally benign, economically effective, and can be scaled up for large-scale synthesis without the use of high pressure, energy, temperature, or harmful compounds. Bioreduction of metal ions employing biomolecules such as enzymes, bacteria, and plant extracts is both ecologically friendly and chemically sophisticated^13^. Among the several green synthesis strategies, plant-mediated synthesis appears to be a promising strategy that allows for faster nanoparticle production and more stable synthesis^14^. The creation of bio-inspired nanoparticles has received much interest, as well as approaches for manipulating nanoparticle size^6,15^.

*Origanum majorana* is a cold-tolerant perennial plant or undershrub with pleasant pine and citrus notes. Marjoram is sometimes confused with oregano in several Middle Eastern nations, and the terms sweet marjoram and twisted marjoram are used to distinguish it from other *Origanum* species. It is sometimes known as pot marjoram^16^, however, this term is also applied to other *Origanum* cultivated species. Soups, stews, salad dressings, sauces, and herbal teas all benefit from the addition of marjoram. Sweet marjoram, also known as *Origanum majorana* L. (*O. majorana*, Lamiaceae family), is a prominent herb applied in traditional medicine for its healing qualities in gastrointestinal, ophthalmic, cardiac, and neurological problems. Significant bioactive elements of *O. majorana* have been identified and isolated, such as volatile compounds, terpenoids, phenolics, flavonoids, and tannins. This herb's ethnopharmacological knowledge revealed that it has antibacterial, antifungal, antiprotozoal, and antioxidant properties. The majority of the treatments are time-consuming, expensive, and necessitate the use of skilled operators and sophisticated instruments. Electrochemical determination approaches, on the other hand, are preferable for determining several biological, environmental, and pharmacological chemicals due to their quick reaction and ease of use^5,17,18^. Nevertheless, oxidizing Tramadol using traditional solid electrodes is a sluggish process that necessitates a larger over-potential. As a result, a simple and sensitive upgraded electrode for quantitative tramadol measurement is required. In contemporary voltammetry, chemically modified electrodes have become a hot topic. The intended analyte measurement becomes more specific and sensitive when these electrodes are used. Nanostructured materials have been used to change electrode surfaces to improve the sensitivity of electrochemical sensors in recent decades^19^. Nanoparticles can be utilized to modify electrodes, allowing for the detection of trace quantities of analytes by improving the sensors' sensitivity and stability^20^. Metal nanomaterials, including transition metal (Co/Ni/Cu) and their oxides^21^, have attracted a lot of attention in past years due to their various advantages of excellent electrocatalytic efficiency, long-term stability, relatively inexpensive, and ease of fabrication and construction of non-enzymatic electrochemical sensors^22^, wherein cupric oxide nanoparticles are favorable in electrocatalytic activity and electrical conductivity, making them an excellent non-enzymatic based electrochemical sensor ingredient^23,24^.

The use of carbon nanotubes in sensors and biosensors has recently received much interest. Because of their exceptional one-dimensional physical and electrical capabilities, multi-walled carbon nanotubes (MWCNTs) are extensively utilized in electroanalytical chemistry^25,26^.

We use the co-precipitation approach to make CuO NPs in this study. In addition, MWCNTs employed to modify a carbon paste electrode. The current study presents a CuONPs/MWCNTs-based tramadol electrochemical sensor that is both selective and sensitive. Finally, real-life samples of Tramadol and acetaminophen analyzed using this modified electrode. Therefore, the current study presents a CuONPs/MWCNTs-based tramadol electrochemical sensor that is both selective and sensitive.

## Materials and methods

### Materials

All of the compounds applied in this study were analytical grade, and they were utilized as-is, with no additional purification. Multi wall carbon nanotubes (MWCNTs) as well as cupric nitrate (Cu(NO_3_)_2_,5H_2_O) was acquired from Merck in Germany for this work. In addition, twofold distilled water (DW) was utilized in all of the tests. A Metrohm 797 was utilized for each electrochemical experiment. In a 10 mL one compartment electrochemical cell, SPE (DropSens; DRP-110) employed three standard electrodes: carbon (4 mm diameter) active electrodes, a graphite counter electrode, and a silver pseudo-reference electrode.

### Green synthesis of CuO nanoparticles

The plant species *Origanum majorana* was obtained from Kerman suburb and validated by a biosystematic plant specialist. A person from herbarium center of Kerman university of medical science assisted us to collect and identify the *Origanum majorana*.

*Origanum majorana* is a cold-sensitive perennial herb or undershrub with sweet pine and citrus flavours. In some Middle Eastern countries, marjoram is synonymous with oregano, and there the names sweet marjoram and knotted marjoram are used to distinguish it from other plants of the genus *Origanum*. It is also called pot marjoram, although this name is also used for other cultivated species of *Origanum*. Marjoram has long been used as a medicinal herb. Marjoram or marjoram oil has been used to treat cancer, colds, coughs, cramps, depression, as a diuretic, ear infections, gastrointestinal problems, headaches, and paralysis, as well as arthritis, chest congestion, and muscle aches. It has also been used as an aphrodisiac, mouthwash, tea, and in poultices, tinctures, and infusions. Though not all of its historic uses are scientifically backed, the plant has verifiable medical use. For example, it contains the phenol carvacrol, which is antibacterial, antifungal and antimicrobial. Ethanol extract is cytotoxic against fibrosarcoma cell lines, ethyl acetate extract has antiproliferative properties against C6 and HeLa cells, as have Hesperetin and hydroquinone, which can be isolated from marjoram extract. Cardioprotective, hepatoprotective, antiulcerogenetic, anticholinesterase, anti-PCOS, and anti-inflammatory effects were also found in dried marjoram, marjoram tea, or in compounds extracted from marjoram. Marjoram is generally not toxic, but should not be used by pregnant or lactating womenHowever, it is always important to be cautious and consult a doctor when using medical herbs^27^.

**Figure 1:**
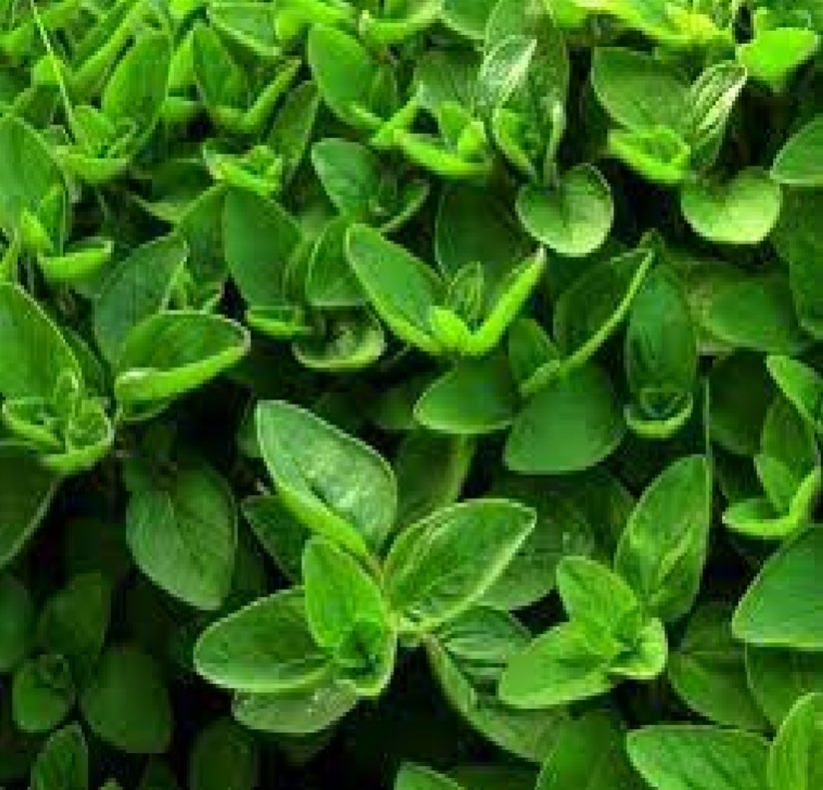
The *Origanum majorana* image.

Figure 1 indicates the *Origanum majorana* image. To make the water based extract, *O. majorana* leaves were first washed with DW water to remove any attached dust particles, then chopped into very small pieces and dried in the sun. After heating 100 mL distilled water to 100 °C, 20 g dried O. majorana leaf powder was added and left to incubate for 10 min. As a result, the supplied leaf extract was allowed to cool at ambient temperature before being filtered using Whatman filter paper. 1 mmol Cu(NO3)02 was diluted in distilled water to a transparent solution to make the CuO nanoparticles. After that, a small quantity of O. majorana extract was added to the mixture and rapidly agitated at 80 °C for 30 min. The resulting products after filtration, were dried for 2 h at 80 °C under vacuum, then calcined for 3 h at 600 °C.

### Preparation of the CuONPs/MWCNTs/carbon paste modified electrode

An Autolab potentiostat/galvanostat was used for the electrochemical experiments, and the General-Purpose Electrochemical System (GPES) software regulates the experimental settings. At 25 ± 1 °C, a standard three-electrode cell was applied. The reference, auxiliary, and working electrodes were an Ag/AgCl/KCl (3.0 M) electrode, a platinum wire, and CuONPs/MWCNTs/carbon paste. A Metrohm 710 pH meter was used to determine the pH. Buffer solutions with a pH range of 2.0–9.0 were made via orthophosphoric acid and its salts. Next, CuONPs/MWCNTs/carbon paste was made by combining 0.01 g MWCNT with 0.95 g graphite powder and 0.04 g CuO nanoparticles in a mortar and pestle by hand. The mixture mentioned above was then combined for 20 min with 0.7 mL paraffin oil until a consistently moistened paste was achieved. After that, the paste was stuffed into the end of a glass tube (ca. 3.4 mm i.d. and 15 cm long), and a copper wire was implanted inside the carbon paste to make the electrical connection.

### Characterization of CuO nanostructures

A range of technologies used to characterize the synthesized samples, including X-ray diffraction (XRD) patterns. The FTIR alpha model of Bruker used to record Fourier transform infrared spectra. In addition, Scanning electron microscopy applied to examine the generated NPs' morphologies. The Vasco model of Nanosizer cordouan used to determine particle size and zeta potential (France). For pH measurements, a Metrohm 827 lab pH meter employed.

### Statistical analysis

Student's t-tests and analysis of variances used to determine group significance. All data provided as mean ± SD. Statistical significance defined as a probability threshold of p = 0.05.

### Theoretical method

The energy of adsorption (Ead) for Tramadol on MWNT calculated using DFT calculations using Guassian 03 software. DFT calculations solely carried out using 6–311 + (d) to minimize computational difficulties and the demand for immense computation potential. All of the buildings that built were geometrically optimized first. The older structures then changed using the geometrically optimized atomic locations, and algorithms run to determine the SCF energies before Ead was eventually calculated.

### Ethical standards

This study was conducted following Compliance with Ethical Standards, and it did not involve human participants, animals, and potential conflicts of interest.

## Results and discussion

### CuO nanostructures characterization

**Figure 2:**
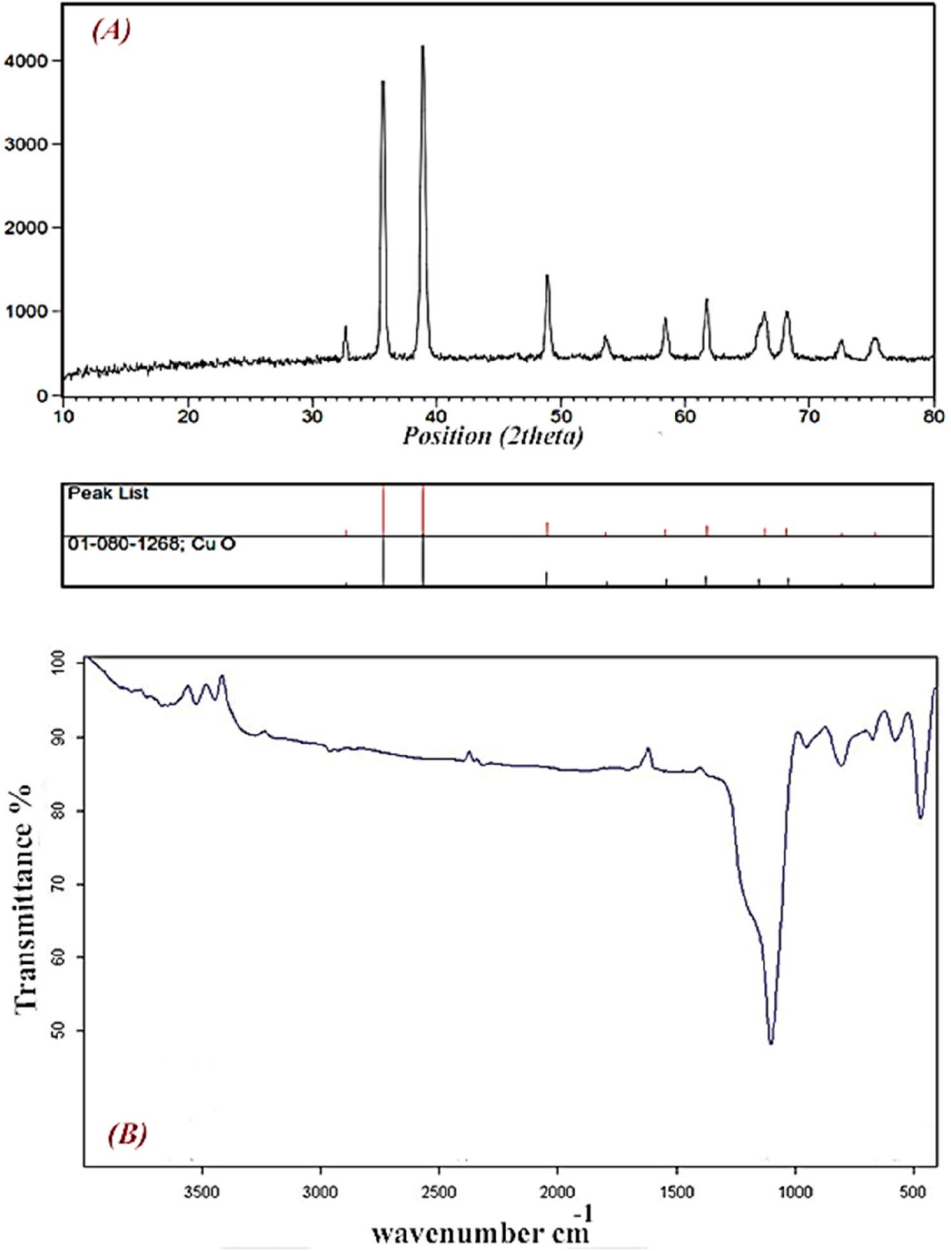
(**A**) XRD pattern and (**B**) FTIR spectra of synthesized CuO nanostructures.

Figure 2A shows the XRD pattern of the produced CuO nanoplates. The generated CuO nanoparticles' diffraction peaks were calculated to be at 2θ values of 32.34° (110), 35.36° (− 111), 38.56° (111), 48.57° (−202), 53.39° (020), 58.14° (202), 61.40° (−113), 66.17° (− 311), 67.98° (220), 72.48° (311) and 75.02° (004), that confirms by CuO (JCPDS 80-1916) and indicated the formation of CuO nanostructures^28^. The crystallite size of CuO nanoparticles was determined to be 38.2 nm utilizing the Deby-Scherer equation^29^. The synthesized nanostructures were pure and no impurities detected.

Figure 2B demonstrates the FTIR spectrum of the synthesized cupric oxide NPs. As seen, the spectrum of the CuO exists in three areas. In the first area, those peaks from 500 to 800 cm^−1^ exhibited a stronger absorption band related to the stretching vibrational of Cu–O vibrations, confirming the synthesis of CuO nanoparticles^30^. However, in the second area (1350 cm^−1^ to 1650 cm^−1^), we may observe peaks due to the presence of CO_2_ in the air. Finally, the third area is between 2800 and 3500 cm^−1^. Therefore, it could be concluded that the hydrated CuO and H_2_O in the air contribute to the peak formation. Therefore, the synthesized CuO NPs present a pure and monolithic phase according to FTIR spectra.

**Figure 3:**
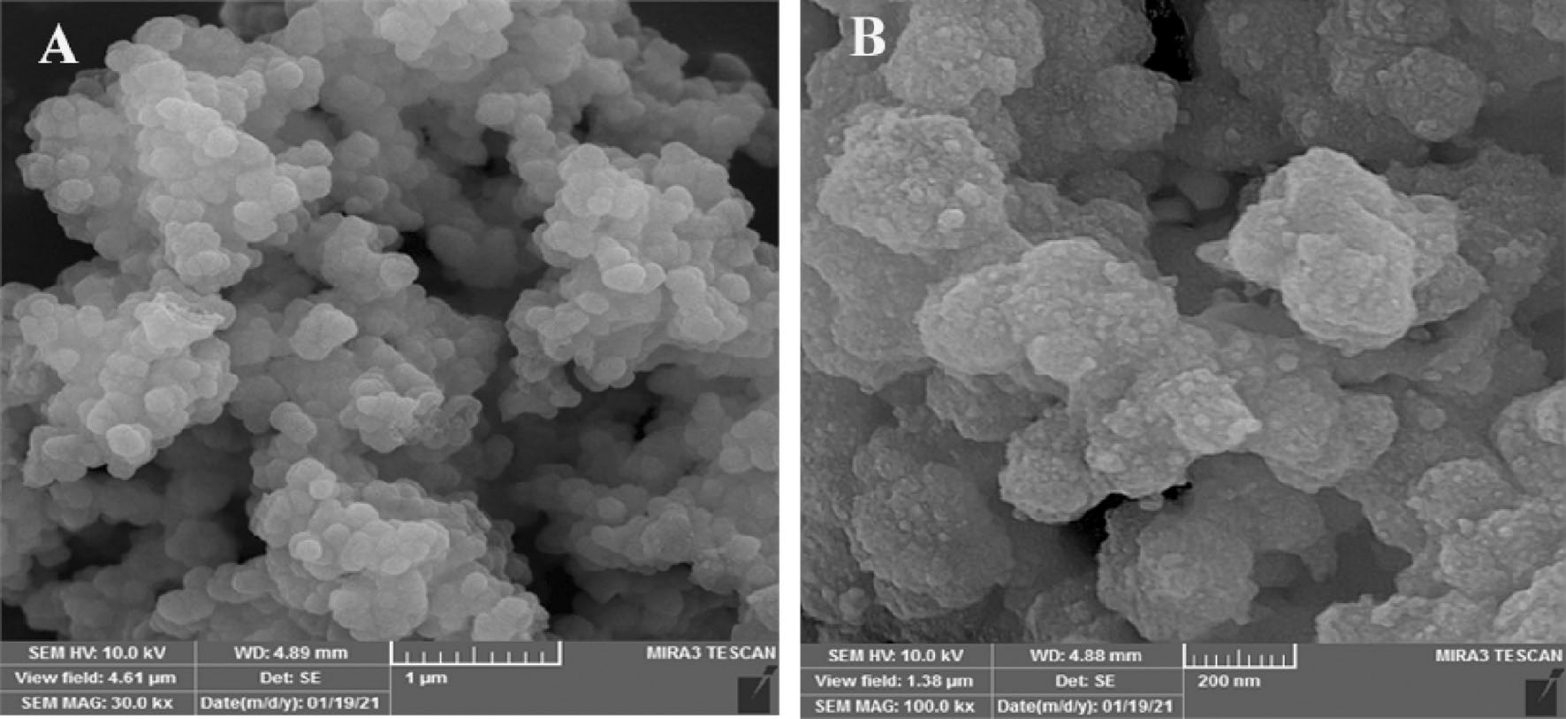
SEM images of synthesized CuO nanostructures.

Figure 3A, B depicts the SEM images of CuO nanoparticles. As presented in Fig. 3, the nanoparticles were uniformly sized and spherical shaped. The size of the particles estimated to be approximately 52 nm. It has been found that the biological synthesis of CuO NPs produces relatively small quasi-spherical particles of homogeneous dimension. The use of biological components in the synthesis process could describe the slight agglomeration in the as-synthesized nanoparticles. The CuO NPs synthesized from leaf extract had a spherical shape, which was consistent with previous findings^31^.

**Figure 4:**
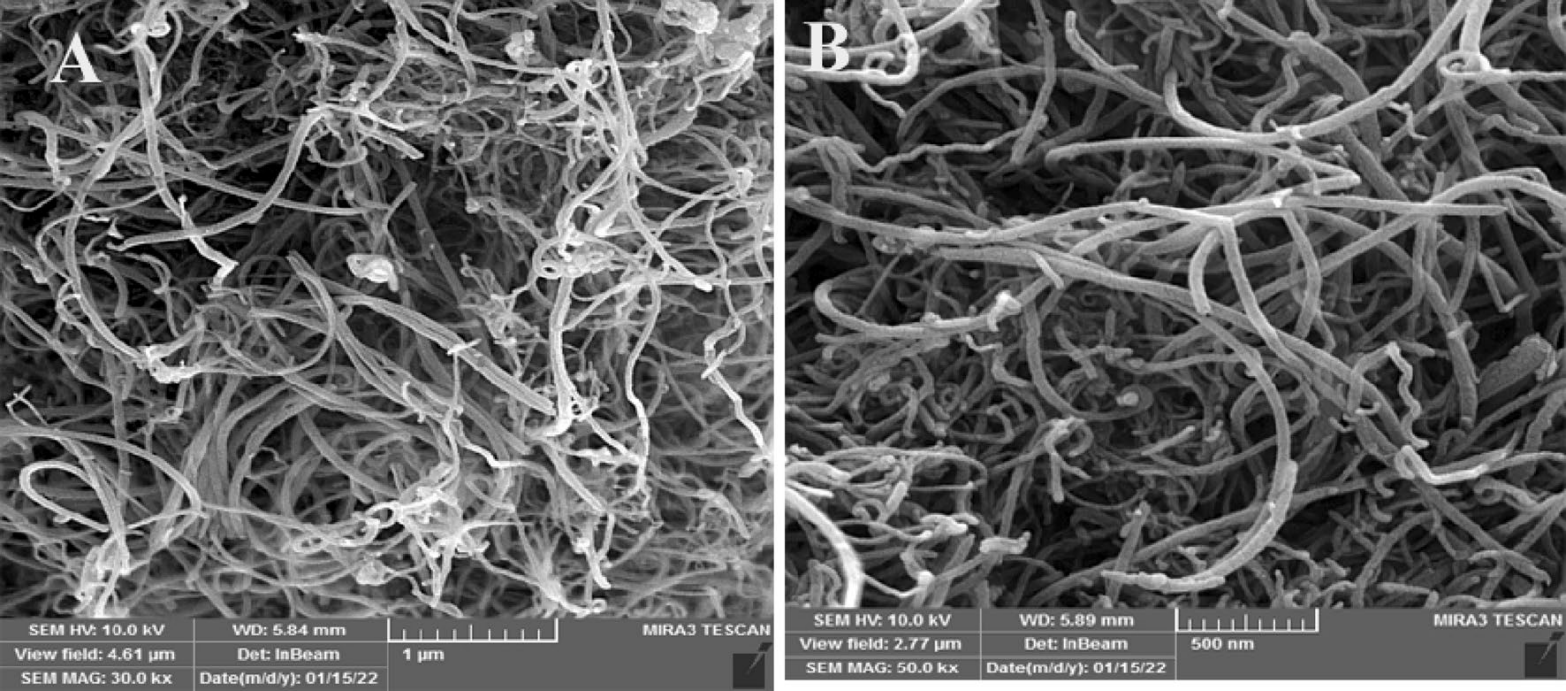
SEM images of used MWCNTs.

Figure 4A, B reviles SEM of multi wall carbon nanotubes. The SEM images show the structural integrity of the CNTs wich have very high MWCNT concentrations.

### CuONPs/MWCNTs/carbon paste electrochemical characteristics

**Figure 5:**
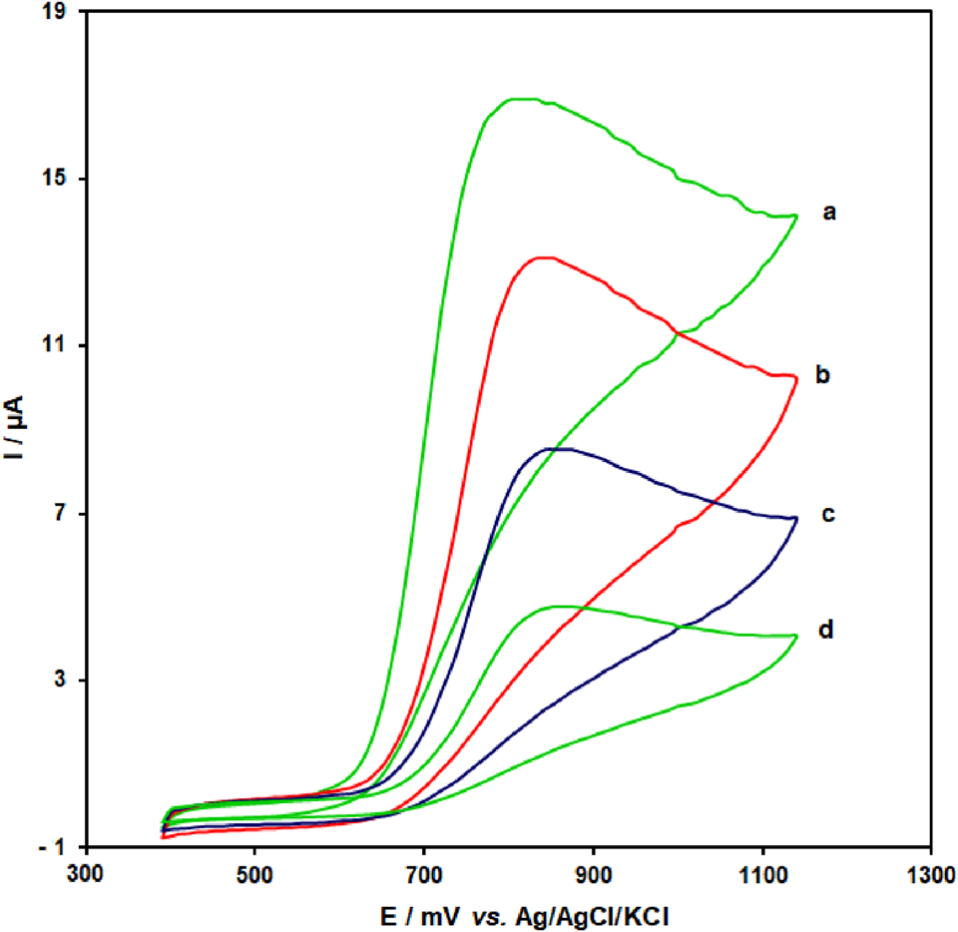
Cyclic voltammograms of (**a**) CuONPs/MWCNTs/CPE, (**b**) MWCNTs/carbon paste, (**c**) CuONPs/carbon paste electrode and (**d**) un-modified carbon paste electrode in the presence of 400.0 μM tramadol at a pH 7.0 of 0.1 M PBS, respectively.

CuONPs/MWCNTs/carbon paste electrochemical sensor examined in 0.1 M PBS (pH 7.0). Figure 5 indicates cyclic voltammograms for CuONPs/MWCNTs/carbon paste of Tramadol; Insets show the linear relationship of the anodic peak current versus square root of the scan rate (v^1/2^).

For CuONPs/MWCNTs/carbon paste in an aqueous solution, the test results reveal anodic and cathodic peaks that are well defined and repeatable with quasi-reversible activity. The CuONPs/MWCNTs/carbon paste's long-term stability also examined over a period of three weeks. Once the reference electrode maintained at 20–22 °C, the maximum potency for tramadol oxidation stayed identical. However, the current signals decreased by b2.4 percent compared to the first response. The improved electrode's antifouling characteristics against tramadol oxidation and its oxidation metabolites examined to evaluate the CVs of the modified electrode before and after application in the corporation of tramadol. CVs obtained after cycling the potential 15 times at a scan rate of 10 mV s^−1^ in the presence of Tramadol. Peak potentials were constant, while currents fell via b2.4 percent. As a result, not only did the sensitivity of the analyte and its oxidation product rise at the surface of CuONPs/MWCNTs/carbon paste, but also the fouling impact reduced as well.

### Electrocatalytic oxidation of tramadol at CuONPs/MWCNTs/carbon paste

**Scheme 1:**
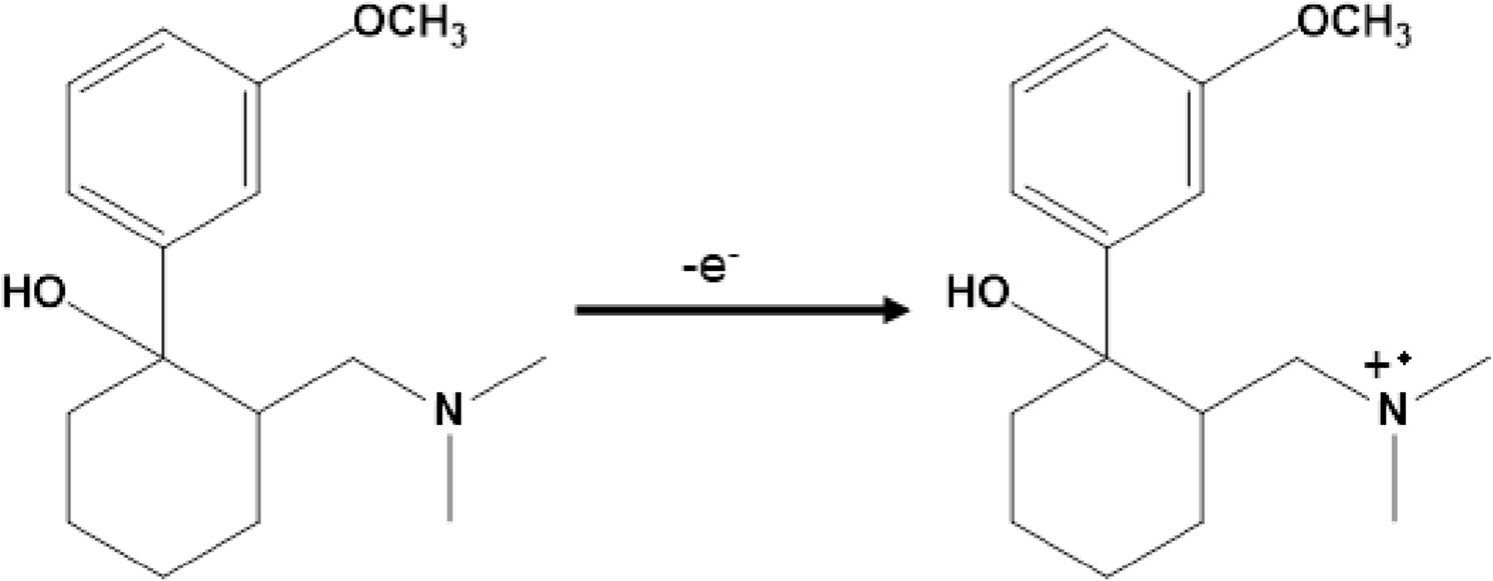
Probable oxidation mechanism for tramadol at CuONPs/MWCNTs/carbon paste.

The aqueous solution's pH level influences tramadol's electrochemical behavior. As a result, pH adjustment of the solution appears to be required for tramadol electrocatalytic oxidation. By CV, the electrochemical activity of tramadol examined at the surface of CuONPs/MWCNTs/carbon paste in 0.1 M PBS at varied pH values (2.0 b pH b 9.0). Under neutral circumstances, the electrocatalytic oxidation of tramadol at the surface of CuONPs/MWCNTs/carbon paste was shown to be more favorable than in an acidic or basic media. In the CVs of CuONPs/MWCNTs/carbon paste, this manifests as a progressive increase in the anodic peak current and a parallel drop in the cathodic peak current. Thus, the optimal pH for tramadol oxidation electrocatalysis at the surface of CuONPs/MWCNTs/carbon paste was found to be 7.0. Scheme 1 depicts the presumed mechanism for oxidation of tramadol.

To investigate the tramadol behavior and also as-produced electrode response to determine tramadol, the performance of CuONPs/MWCNTs/CPE was compared to that of MWCNTs/CPE, CuONPs/CPE, and unmodified CPE. Figure 5 shows the CV curves obtained for CuONPs/MWCNTs/CPE (curves a), MWCNTs/CPE (curves b), CuONPs/CPE (curves c) unmodified CPE (curves d) in the presence of 400.0 μM tramadol-containing 0.1 M PBS at the scan rate of 50 mV/s.

The anodic peak potentials for tramadol oxidation at CuONPs/MWCNTs/carbon paste and unmodified CPE are 875 and 915 mV, whereas the equivalent potential at CuONPs/MWCNTs/carbon paste is 655 mV. These findings show that when compared to CuONPs/MWCNTs/carbon paste and unmodified CPE, the maximum value for tramadol oxidation at the CuONPs/MWCNTs/carbon paste electrodes shifts by 220 and 260 mV in the direction of negative values. CuONPs/MWCNTs/carbon paste, on the other hand, has a substantially greater anodic peak current for tramadol oxidation than CuONPs/MWCNTs/carbon paste.

### Effect of scan rate

**Figure 6:**
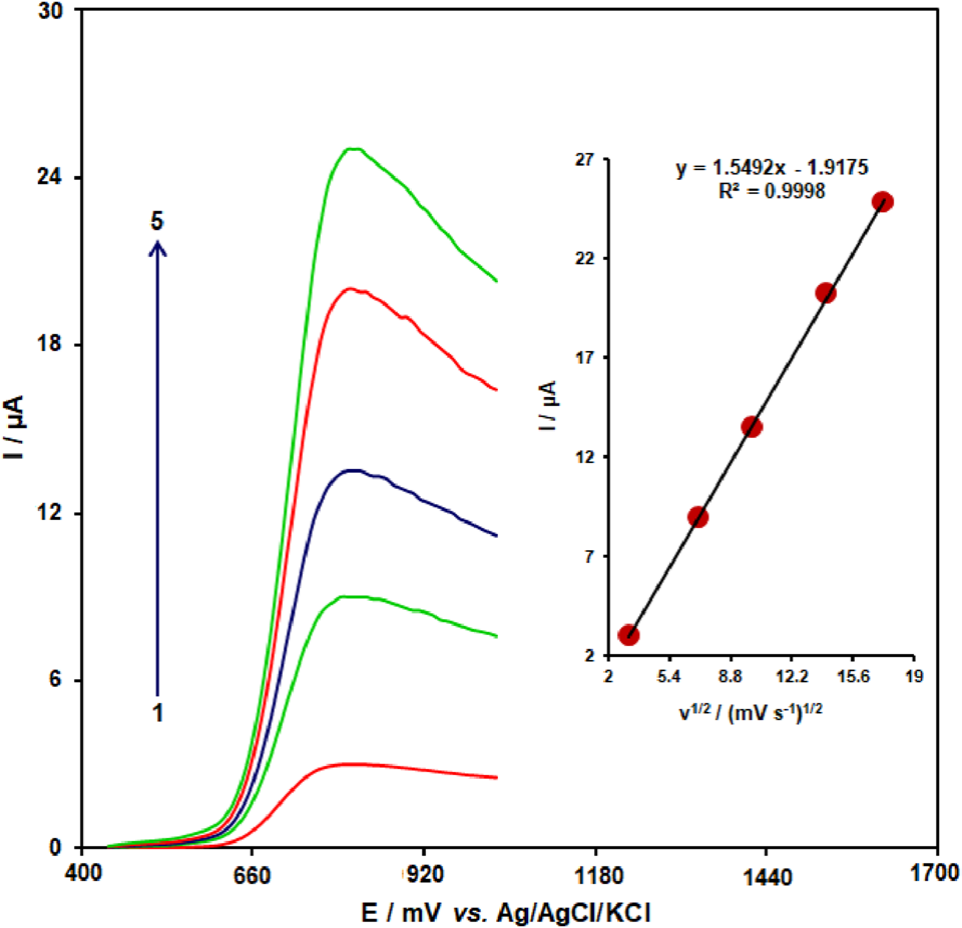
Linear sweep voltammograms of tramadol (400.0 μM) at CuONPs/MWCNTs/CPE at different scan rates of 1, 2, 3, 4, 5, and 6 mV/s in 0.1 M PBS (pH 7.0). Insert: plot of *I*p versus *ν*^1/2^ for the oxidation of tramadol at CuONPs/MWCNTs/CPE.

The linear sweep voltammograms measurements were carried out to evaluate the association of peak current with scan rate at varied scan rates (10–400 mV/s) in the 400.0 μM tramadol-containing 0.1 M PBS (pH  7.0) on the CuONPs/MWCNTs/CPE (Fig. 6). As shown in Fig. 6, the peak currents of tramadol grow with increasing scan rates and there are good linear relationships between the peak currents (*I*p) and square root of the scan rate (*ν*^1/2^). The results also showed that the action is mass transfer of tramadol controlled at diffusion process.

### Chronoamperometric measurements

**Figure 7:**
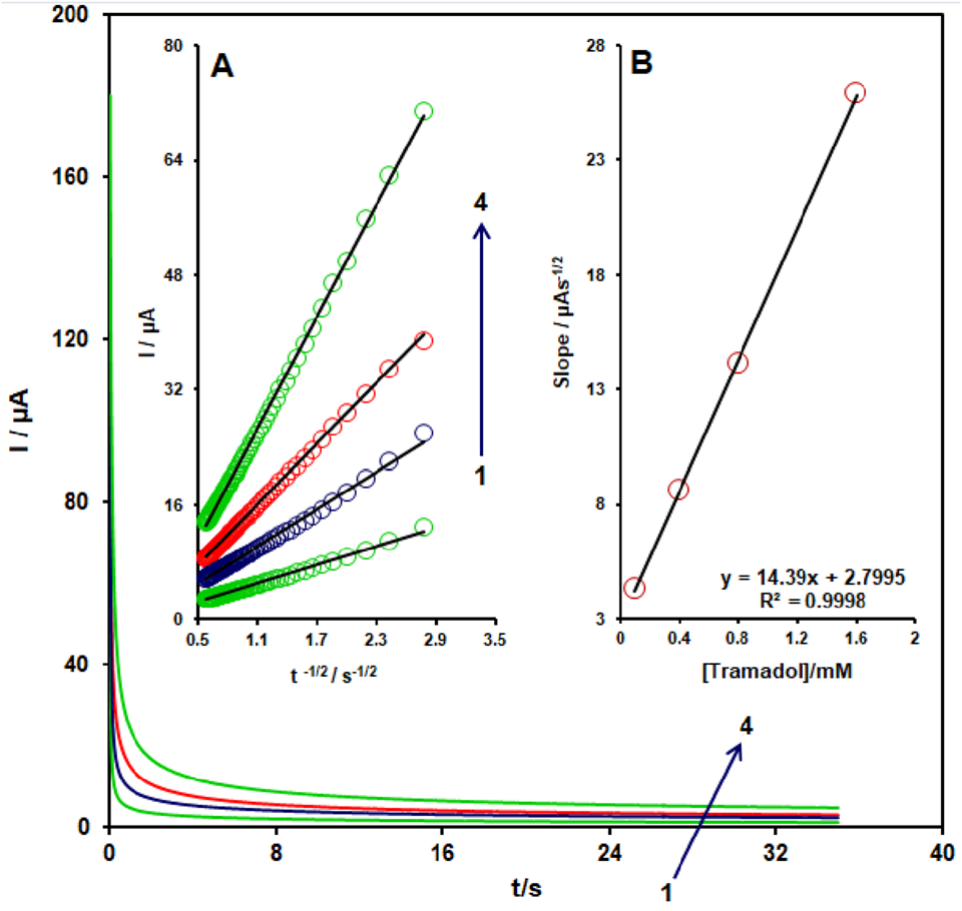
Chronoamperograms obtained at the CuONPs/MWCNTs/CPE in the presence of (1) 3.0, (2) 6.0, (3) 9.0, and (4) 1.0 mM tramadol in the0.1 M buffer solution (pH 7.0). (**A**) Plot of *I* versus *t*^-1/2^ for electro-oxidation of tramadol obtained from chronoamperoms 1–4. (**B**) Plot of slope from straight lines versus tramadol level.

For the different doses of Tramadol in 0.1 MPBS (pH 7.0), chromatoamperometric measurements of Tramadol at CuONPs/MWCNTs/carbon paste were accomplished by placing the working electrode potential at 0.70 V (at the first potential step) and 0.40 V (at the second potential step) (Fig. 7). Using chronoamperometric studies, we determined the diffusion coefficient, *D*, of tramadol in buffer solution.

For an electroactive drug (tramadol in this case) with a diffusion coefficient of D, the Cottrell equation outlines the current observed for the electrochemical process underneath the mass transportation limited state.

The best fits for varied tramadol doses were found using experimental plots of I vs. t − 1/2. The slopes of the straight lines resulted plotted upon the tramadol level.The average rate of the D found to be 6.85 × 10^−6^ cm^2^/s using the resultant slope and Cottrell equation.

### Limit of detecting and calibrating curve

**Figure 8:**
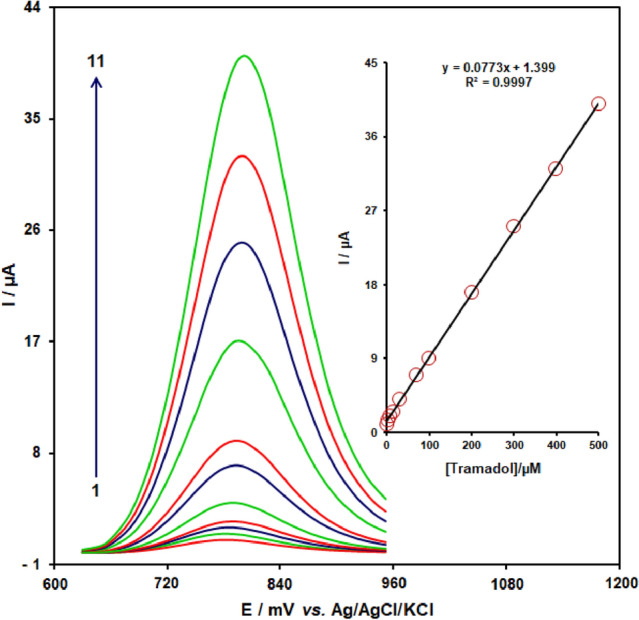
SWV curves of CuONPs/MWCNTs/CPE in the0.1 M buffer solution (pH 7.0) containing different concentrations of tramadol. Inset: Plots of electro-catalytic peak current as a function of tramadol concentration.

The tramadol concentration was determined using the square wave voltammetry (SWV) technique (Fig. 8). Two linear segments with slopes of 0.7441 and 0.1378 μA μM—made up the plot of peak current vs. tramadol dosage. The kinetic restriction is most likely to blame for reducing the second linear segment's sensitivity. The tramadol detection limit (3σ) was 25 ± 2 nM. This number is comparable to tramadol determinations at the exterior of chemically altered electrodes published via similar research groups.

Hence, Table 1 shows that the CuONPs/MWCNTs/carbon paste can compete with other sensors for the determination of tramadol.

**Table 1:** Comparison the sensing performances toward the detection of tramadol between the existing modified electrodes and the proposed CuONPs/MWCNTs/carbon paste.

Electrochemical sensor	Method	Linear range	LOD	Ref
Magneto layer double hydroxide (LDH)/Fe_3_O_4_modifiedglassy carbon electrode	Differential pulse voltammetry	1.0–200.0 μM	0.3 μM	^32^
Poly(Nile blue) modified glassy carbon electrode	Differential pulse voltammetry	1.0–310.0 μM	0.5 μM	^33^
Gold nanoparticles/cysteic acid modified glassy carbon electrode	SWV	0.50–63.6 μM	0.17 μM	^34^
Multi-walled carbon nanotube-modified glassy carbon electrode	Differential pulse voltammetry	2.0–300.0 μM	0.361 μM	^35^
CuONPs/MWCNTs/CPE	SWV	0.05–200.0 µM	25 ± 2 nM	This work

Figure 8 indicates SWVs for CuONPs/MWCNTs/carbon paste in 4 mmol L^−1^ tramadol at various pHs (pH 5.5, 7, 8.5, 10) (d to a).

### The stability of the response at the modified electrode

The stability of the CuONPs/MWCNTs/CPE was examined by storing the electrode in the lab at room temperature. Then, the electrode was used for the analysis of 50 μM of tramadol from 1 to 21 days intervals in 0.1 M PBS (pH 7.0). The results showed that the electrode signal retained to 92% of its initial value after 7 days and 90% of its initial value after 21 days. these results indicated that the proposed electrochemical sensor had excellent long-term stability.

### Computational method

**Figure 9:**
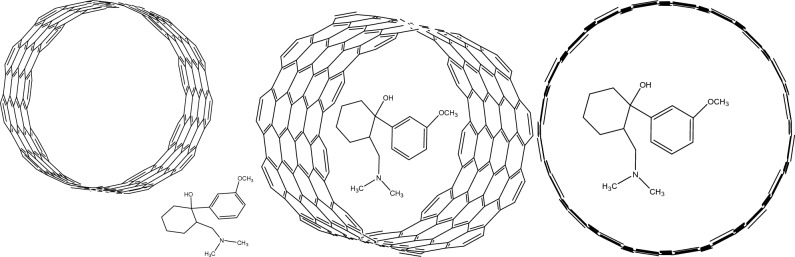
Different view of Tramadol on and inter of MWNT.

**Figure 10:**
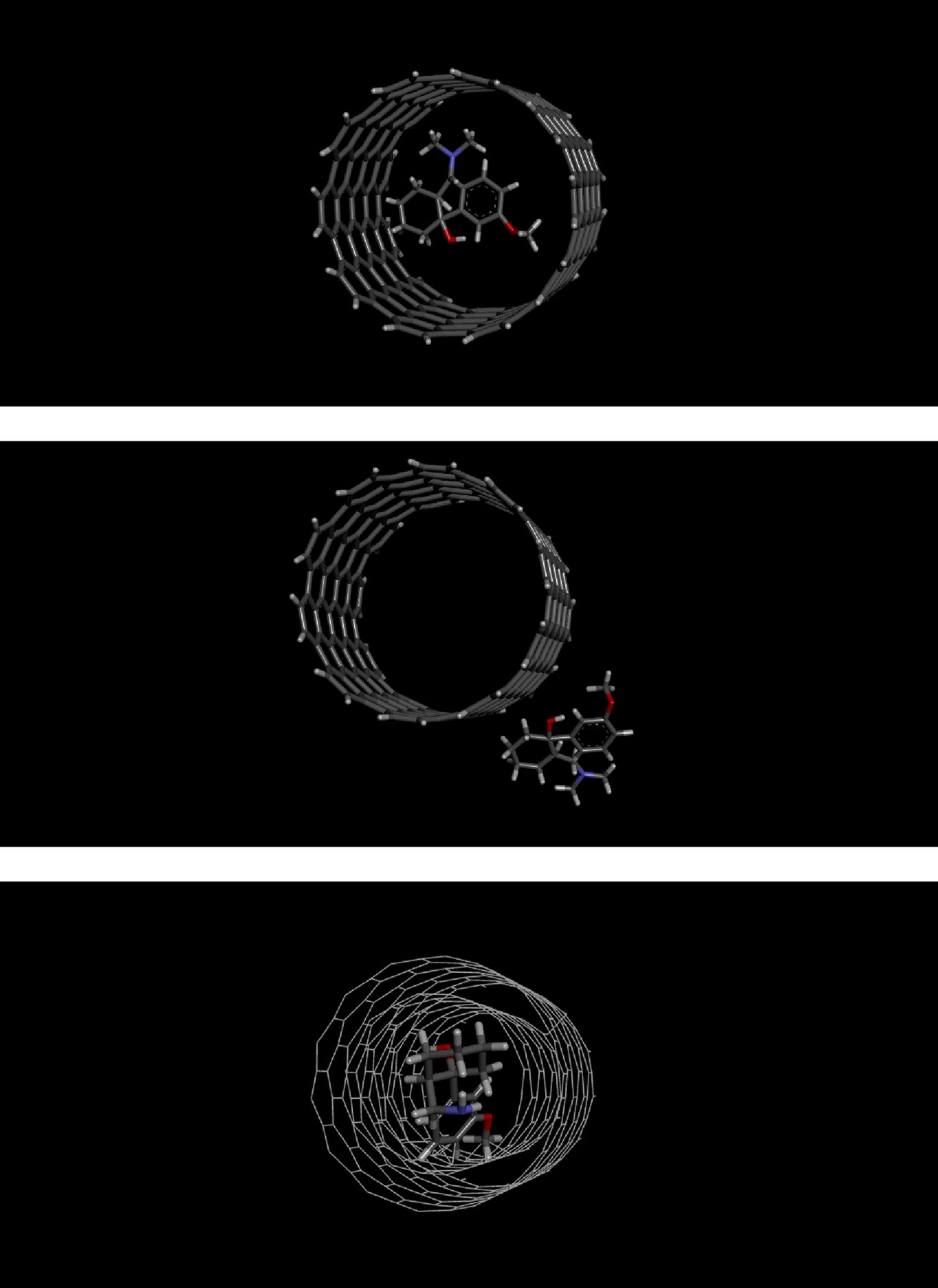
Different view of Tramadol on and inter of MWNT.

The energy of adsorption (Ead) for Tramadol on MWNT calculated using DFT calculations using Guassian 03 software^36^. The value of Ead as calculated for tramadol adsorption on the MWNT was 5.06 × 10^–19^ kcal and 4.94 × 10^–19^ kcal on and inter of MWNT, respectively. However, depending on the DFT input parameters used, Ead values can vary greatly, and Ead values can also fluctuate for different poses of an adsorbent for a particular adsorptive^37^. The Ead sign is frequently used to determine whether an adsorption process is exothermic or endothermic. A negative sign in the formula for calculating Ead denotes an endothermic reaction. Thus, the DFT calculations, which agree with the experimental results, also point to the endothermic character of the adsorption mechanism (to be more specific, the DFT calculations point to the endothermic feature of Tramadol adsorption on the MWNT). Figure 9 indicates different view of Tramadol on and inter of MWNT and Fig. 10 shows various view of Tramadol on and inter of MWNT.

## Conclusions

The use of *Origanum majorana* as alkaline agent in the green production of CuO nanostructures was described in this study. One of the innovative materials employed for tramadol determination was a CuONPs/MWCNTs/carbon paste modified electrode. CuONPs/MWCNTs nanocomposite improved tramadol oxidation selectivity and electrochemical activity. The linear calibration curve in ranges between 0.07 and 300 µM with a LOD of 0.01 µM for MO was produced using the optimal condition. Finally, the modified electrode substantially used for tramadol analysis in the real specimens. The proposed method offers a sensitive approach to detect tramadol in drug and biological formulations. Furthermore, this modified electrode may be used to identify tramadol in human plasma and urine and also drug samples.

## Data Availability

The datasets used and/or analyzed during the current study available from the corresponding author on reasonable request.
